# Obesity and climate adaptation

**DOI:** 10.1093/emph/eoz016

**Published:** 2019-05-31

**Authors:** Diego Salazar-Tortosa, Lindsay Fernández-Rhodes

**Affiliations:** 1 Department of Ecology, University of Granada, Avenida de Fuentenueva s/n, Granada 18071, Spain; 2 Department of Biobehavioral Health, Pennsylvania State University, 219 Biobehavioral Health Building, University Park, PA 16802, USA

## OBESITY

Obesity is a pandemic that has increased exponentially during the past decades due in large part to recent changes in lifestyle and food delivery systems. Yet, there is still a great variability in the burden of obesity across ancestral populations. For example, in the USA prevalence estimates of adult obesity vary from 13 to 48% in Asian Americans and African Americans.^1^ Such variable observations within the same obesogenic environment have motivated a wide array of inquiry into the genetic, epigenetic and social determinants of obesity, and their complex interactions with modern lifestyles and food systems.

## EVOLUTIONARY PERSPECTIVES

**Figure 1.**
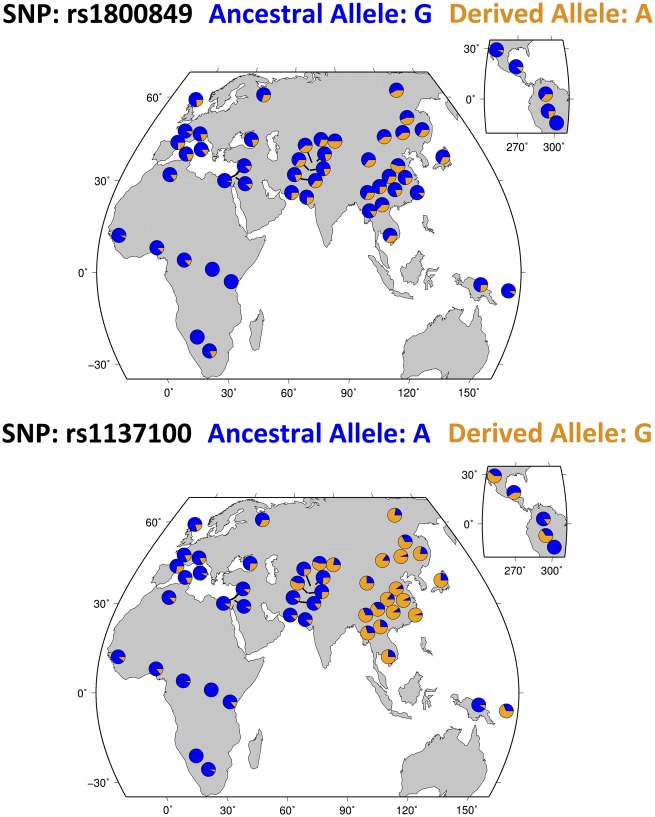
Geographical distribution of rs1800849 at UCP3 and rs1137100 at LEPR single nucleotide polymorphisms (SNPs; adapted from the selection browser of the Human Genome Diversity Panel^8^)

Variation in obesity burden across ancestral populations may be partially explained by human history. After migrating out of Africa, humans were exposed to a broader range of environmental conditions. For example, exposure to colder climates may have shaped human metabolism by positively selecting for a higher resting metabolic rate (RMR), at the expense of building lower adipose stores for times of food insecurity.^2^ This evolutionary trade-off has been supported by the fact that RMR varies between populations, being highest in indigenous-arctic populations, intermediate for European ancestry and lowest in African Americans.^3^^,^^4^ This could partially explain why certain ancestral groups within the same country, e.g. European Americans or East Asian Americans, have a lower obesity burden.^5^ In fact, there is evidence of selection by climate across the genome,^6^ and specifically in genes related to the non-shivering thermogenesis of brown adipose tissue (BAT) like those encoding the uncoupling proteins (i.e. *UCP1-3*) or the leptin receptor (*LEPR*).^4^^,^^7^ As shown in Fig. 1 for rs1800849 at *UCP3* and rs1137100 at *LEPR*, adaptation to the colder environmental conditions of high latitudes may have selected for a higher RMR in these populations, as compared with populations with a higher frequency of the ancestral alleles that protect against overheating and promote greater storage of excess energy.

## FUTURE IMPLICATIONS

The complex genetic architecture of obesity has been extensively studied by genome-wide association studies. Yet, additional insights on how the frequency of obesity-related variants was shaped by selective pressures and further modified by possibly heritable epigenetic processes are needed to form a more integrated view of the complex origins of obesity. For example, DNA methylation sites are enriched in regions of the genome with signals of natural selection.^9^ Both genetic variation and epigenetic processes have been implicated in *UCP1* expression in BAT in response to cold exposure.^10^ Prediction models of thermogenesis-dependent expression and protein–protein interactions, along with the search for selection signatures could identify strong obesity candidate genes for future study. This could improve our knowledge of the genetic basis of differential obesity susceptibility and move clinical care closer to the goal of precision medicine. Evolutionary principles may inform the prevention and treatment of obesity via the development of tailored lifestyle guidelines. For example, caloric intake recommendations may want to take differences in RMR across ancestral populations into account.^5^ Thus, taking an evolutionary perspective to the study of obesity may shed light on the role of evolutionary trade-offs and mismatches in both clinical care and global health.
